# A Case of Incontinence of Urine

**Published:** 1803-11-01

**Authors:** R. Cooke


### A CASE OF INCONTINENCE OF URINE; *communicated by* MR. R. COOKE.

THERE has occurred to me of late several cases of persons afflicted with incontinence of urine, which evidently appeared to be occasioned from *blows* received on the loins, and which have baffled every medicine which has been given to *relieve*.

P. L. aged 38, a strong, healthy man, received, while at work, a violent blow across the loins from a truck, which almost immediately caused his urine to flow involuntary. At first, there was considerable symptomatic fever, for which he was ordered saline medicines; the bowels were kept open, and at night an opiate was given. The next day a blister was applied across the loins, and the fever subsiding, the tinct. cantharid. was administered, afterwards alum, camphor, volatiles, opium, zinci, calcin, &c. &c. but none of them had the least influence over the complaint. Electricity and cold bathing were also employed, but with no better effect. The sal martis was given, and after this the bark with valerian, but to no good purpose, except that of improving the man's general health.

The pain in his back, which he originally so much complained of, is in a great measure removed, but his mind is made miserable with the reflection of his never again being capable of following his usual employ.

Last week I had an opportunity of seeing another case very similar to the above, and which has every appearance of proving equally distressing.

My only motive for requesting the insertion of the above is in hopes that some useful observations may be thrown out respecting the treatment of such a very formidable and unmanageable disease.

*London, Oct.* 11, 1803.

